# Periodontal impact in acute myocardial infarction: A comparative study using Russell's periodontal index

**DOI:** 10.6026/973206300221332

**Published:** 2026-03-31

**Authors:** Nidhi Dhakray, Dushyant Pippal, Bhavna Jha Kukreja, Surbhi Priyadarshi, Shubhangi Tiwari, Arun Kumar Chourasia, Santosh Kumar

**Affiliations:** 1 Department of Periodontics and Oral Implantology, Maharana Pratap College of dentistry and research centre, Gwalior, Madhya Pradesh, India; 2 Department of Periodontology, Mansarovar Dental College, Bhopal, Madhya Pradesh, India; 3 Department of Preventive Dental Sciences, College of Dentistry, Gulf Medical University, Ajman UAE; 4 Department of Public Health Dentistry, Faculty of Dental Sciences, SGT University, Gurugram, Haryana, India; 5 Department of Periodontics and Oral Implantology, New Horizon Dental College and Research Institute, Bilaspur, Chhattisgarh, India; 6 Department of Periodontology & Implantology, Karnavati University, Uvarsad, Gandhinagar, Ahmedabad, Gujarat, India

**Keywords:** Periodontal disease, acute myocardial infarction (AMI), Russell's periodontal index (RPI), cardiovascular risk, systemic inflammation

## Abstract

Acute myocardial infarction (AMI) represents a leading global cause of mortality, with emerging evidence linking periodontal disease to cardiovascular risk through systemic inflammatory pathways. Hence, this comparative cross-sectional study evaluated periodontal status using the Russell Periodontal Index (RPI) in 90 AMI patients versus 90 age- and gender-matched healthy controls. AMI patients exhibited significantly higher mean RPI scores (4.82 ± 1.94) compared to controls (1.76 ± 1.23; p < 0.001), with 71.1% showing moderate-to-severe periodontal disease. Multivariate regression analysis confirmed severe periodontal disease as an independent AMI predictor (OR = 2.89; 95% CI: 1.67-4.98) after adjusting for conventional cardiovascular risk factors. These findings advance cardio-periodontology by establishing oral health assessment as a critical component of comprehensive cardiovascular risk stratification and preventive care.

## Background:

Cardiovascular diseases (CVDs) are the leading cause of death on the global level, with some 17.9 million deaths each year, with acute myocardial infarction (AMI) being among the most serious forms of the disease [1]. In spite of the important progress in the field of therapeutic intervention and preventive methods, the CVD burden keeps increasing and it is necessary to identify new risk factors and risk determinants that can lead to the disease's pathogenesis [2]. The conventional cardiovascular risk factors, such as hypertension, diabetes mellitus, dyslipidemia, obesity and smoking, have been well defined, but still, they do not provide a comprehensive explanation of the development of cardiovascular events in a significant percentage of patients [3]. Periodontal disease is a chronic inflammatory disease in the supporting structures of the dentition, of which much attention has been paid as a possible cause of systemic inflammation and atherosclerotic cardiovascular disease [4]. Periodontal disease is among the most common forms of chronic inflammatory diseases in the world, with a prevalence rate of 20-50 per cent in the adult populations [5]. The pathophysiology of periodontal disease is that of the accumulation of the biofilm of bacteria on the surfaces of teeth, which causes host immune reaction, but when not regulated, causes progressive destruction of periodontal tissues [6]. Several mechanistic pathways indicate the biological plausibility of a case in which periodontal disease is associated with cardiovascular pathology. Bacterial periodontal pathogens such as *Porphyromonas gingivalis, Aggregatibacter actinomycetemcomitans*and *Tannerella forsythia* have been identified in atherosclerotic plaques, which indicate direct attacks on vascular tissues by bacteria [7]. Moreover, systemic release of inflammatory mediators, interleukin-1b, interleukin-6, tumour necrosis factor-A and C-reactive protein, released by inflamed periodontal tissues, are associated with endothelial dysfunction, lipid metabolism changes and procoagulant conditions, which favour atherothrombotic events [8]. Similar epidemiological studies have repeatedly shown the relationships between periodontal disease and several cardiovascular events. Reports on meta-analyses indicate that people with periodontal disease have an increased risk of coronary heart disease of 1.2/ 1.5 fold higher than those with a healthy periodontium [9].

Potential cohort studies have also found that periodontal disease is a precursor to cardiovascular events, which lends a time effect in the causal relationship, which is causal in nature [10]. But the inconsistency in the periodontal assessment techniques among studies has inhibited the generalizability and comparability of results. The Russell Periodontal Index (RPI) is one of the first standardised indices that are used to assess the severity of periodontal disease epidemiologically [11]. It is an index that measures the presence and degree of gingival inflammation, formation of periodontal pockets and masticatory function loss, which gives a holistic approach to periodontal status in an ordinal scale [12]. Since the emergence of more modern indices, RPI is still useful in population-based research because of its simplicity, reproducibility and capacity to obtain the range of the severity of periodontal diseases. Several studies have been conducted to determine the association between periodontal indices and cardiovascular disease with different outcomes. Coronary artery disease severity has been found to have strong associations with studies that employ the Community Periodontal Index of Treatment Needs (CPITN) [13]. Nevertheless, few studies have utilised RPI specifically in the acute coronary syndromes and this is a significant gap in the literature. Moreover, most studies have been performed in the Western population and the number of research studies in various geographical and ethnic settings is rather small [14]. The periodontal-cardiovascular relationship can also be explained using the inflammatory hypothesis of atherosclerosis. Chronic low-grade inflammation, which is indicated by the high level of inflammatory biomarkers, is central in every step of atherogenesis, including endothelial activation, to plaque rupture [15]. Periodontal disease, with its unremitting bacterial attack and inflammatory tissue fading, can be one of the critical contributors to systemic inflammatory load, thus accelerating atherosclerotic development and promoting the occurrence of acute cardiovascular events [1]. Therefore, it is of interest to evaluate and compare the periodontal status of patients with acute myocardial infarction and healthy controls using the Russell Periodontal Index and to examine the correlation between the severity of periodontal disease and AMI after controlling for other conventional cardiovascular risks.

## Materials and Methods:

## Study design and setting:

The study was a comparative cross-sectional study carried out at the Cardiac Care Unit and Department of Periodontology of a tertiary care hospital between January 2023 and December 2023. The procedure was conducted following the Declaration of Helsinki and the board of the Ethics Committee of the institution approved the study protocol. All participants were asked to provide written informed consent before enrolling themselves.

## Sample size calculation:

The sample size was determined by the past research that has reported periodontal indices differences between cardiovascular disease patients and healthy controls. Using the assumptions of a mean difference of 1.5 between groups in RPI scores, a standard deviation of 2.0, an alpha error of 0.05 and a power of 80, the required sample size was 72 participants in each group. In order to facilitate the consideration of those who might drop out and those who might not have finished the data, 90 individuals were recruited in each group, resulting in a total of 180 individuals.

## Study population:

## Case group:

The Cardiac Care Unit recruited 90 patients who had an acute myocardial infarction. The diagnosis of AMI was made using the Fourth Universal Definition of Myocardial Infarction criteria, which included the detection of high cardiac troponin levels exceeding the 99 th percentile upper reference limit and at least one of the following: symptoms of acute myocardial ischemia, new ischemic electrocardiographic changes, the appearance of pathological Q waves or imaging evidence of new loss of viable myocardium or regional wall motion abnormality.

## Control group:

The patient population of the general medicine outpatient department was used to recruit 90 age and gender matched healthy controls, who visited the department to get routine health check-ups. The control was also assured of the lack of cardiovascular disease according to the clinical history, physical examination, electrocardiography and echocardiography.

## Inclusion and exclusion criteria:

## Inclusion criteria:

[1] Age between 35 and 70 years

[2] A minimum of 15 natural teeth present.

[3] Capability of giving informed consent.

[4] Cases: confirmed AMI (72 hours of admission) diagnosis.

## Exclusion criteria:

[1] Periodontal treatment during the last 6 months.

[2] In the last 3 months, antibiotic = no antibiotics used.

[3] Pregnancy or lactation

[4] Immunocompromising diseases (HIV/ AIDS, malignancies, immunosuppressive therapy)

[5] Chronic kidney disease (eGFR < 30 mL/min/1.73m 2)

[6] Chronic liver disease

[7] Autoimmune disorders

[8] Edentulous patients

## Data collection:

## Demographics and medical history:

The data on age, gender, educational status and occupation, smoking history (never, former, current), alcohol use and medical history (hypertension, diabetes mellitus and dyslipidemia) were collected using structured questionnaires. The height and weight were measured and used to compute the body mass index (BMI).

## Periodontal examination:

A single trained and calibrated examiner was used to perform periodontal assessment to reduce inter-examiner variability. The reliability was intra-examiner based on the duplicate examination of 20 patients and the kappa coefficient was found to be 0.89. The assessment was conducted with the help of a mouth mirror and Williams's periodontal probe with a sufficient amount of light. The Periodontal Index by Russell was used on each tooth present without exception of third molars.

The criteria used in the index were:

[1] Score 0: Negative - there is no apparent inflammation or dysfunction.

[2] Score 1: Mild gingivitis - open inflammation of the free gingiva, without surrounding the tooth.

[3] Score 2: Gingivitis -inflammation entirely encircles the tooth and there is no evident rupture of the epithelial attachment.

[4] Get 6: A case of gingivitis with pocket formation - broken epithelial attachment, no interference with masticatory function, no mobility or drifting of teeth.

[5] Score 8: Advanced destruction - severe alveolar bone loss, masticatory dysfunction, tooth may be loose, drifted or depressible.

The RPI score was calculated at the individual level as the total number of scores of all the teeth checked divided by the number of teeth checked. The respondents were divided into periodontal severity: healthy/mild (RPI 01.9), moderate (RPI 2.0 4.9) and severe (RPI 5.0 8.0) groups.

## Laboratory parameters:

In the case of AMI patients, regular laboratory tests such as complete blood count, lipid profile, fasting blood glucose, serum creatinine and cardiac biomarkers (troponin I, creatine kinase-MB) were obtained from medical records. The controls were lipid profile and fasting blood glucose.

## Statistical analysis:

Data were processed with the help of Microsoft Excel and analysed with SPSS version 26.0 (IBM Corporation, Armonk, NY). Continuous variables were given in the form of mean plus standard deviation (SD) and categorical variables in the form of frequencies and percentages. The Shapiro-Wilk test was used to test the normality of the continuous variables. Independent samples t-test and Mann-Whitney U test were used to compare the continuous variables between the groups in case of normally distributed and non-normally distributed data, respectively. Comparison of categorical variables was done through the Chi-square test or Fisher's exact test as needed. The multivariate logistic regression analysis was conducted to determine the independent relationship between the periodontal disease severity and AMI, controlling for the potential confounding variables (age, gender, smoking, hypertension, diabetes mellitus, dyslipidemia and BMI). Odds ratios (OR) with confidence intervals (CI) were obtained. The level of statistical significance was determined as p < 0.05.

## Results:

A total of 180 participants were enrolled in the study, with 90 participants in each group. The demographic and clinical characteristics of the study population are presented in Table 1 (see PDF). The mean age of AMI patients was 54.8 ± 9.2 years, compared to 53.6 ± 8.7 years in the control group (p = 0.372). Males constituted 73.3% of the AMI group and 70.0% of the control group (p = 0.613). Significant differences were observed between groups regarding conventional cardiovascular risk factors. The prevalence of hypertension was significantly higher in AMI patients (62.2%) compared to controls (31.1%) (P<0.001). Similarly, diabetes mellitus was more prevalent among AMI patients (44.4%) than controls (17.8%) (P<0.001). Current smoking was reported by 46.7% of AMI patients versus 23.3% of controls p = 0.001). Mean BMI was significantly higher in the AMI group (27.4 ± 3.8 kg/m^2^) compared to controls (25.1 ± 3.2 kg/m^2^) (p < 0.001). Periodontal parameters assessed using Russell's Periodontal Index are summarised in Table 2 (see PDF). The mean RPI score was significantly higher in AMI patients (4.82 ± 1.94) compared to controls (1.76 ± 1.23) (p < 0.001). When categorised by severity, 71.1% of AMI patients exhibited moderate to severe periodontal disease, compared to only 23.3% of controls (p < 0.001). Among AMI patients, severe periodontal disease (RPI ≥ 5.0) was observed in 38.9% of participants, compared to 6.7% in the control group. Healthy or mild periodontal status (RPI < 2.0) was present in only 28.9% of AMI patients, whereas 76.7% of controls fell into this category. The distribution of individual RPI scores revealed that scores of 6 (gingivitis with pocket formation) and 8 (advanced destruction) were significantly more frequent among AMI patients. Specifically, 58.9% of AMI patients had at least one tooth with an RPI score of 8, compared to 14.4% of controls (p < 0.001). Multivariate logistic regression analysis was performed to assess the independent association between periodontal disease severity and AMI (Table 3 - see PDF). After adjusting for age, gender, smoking status, hypertension, diabetes mellitus, dyslipidemia and BMI, moderate to severe periodontal disease (RPI ≥ 2.0) remained significantly associated with AMI (adjusted OR = 2.89, 95% CI: 1.67-4.98, p < 0.001). When analysed by periodontal severity categories, participants with moderate periodontal disease had 2.42 times higher odds of having AMI compared to those with healthy/mild periodontal status (95% CI: 1.18-4.96, p = 0.016). Participants with severe periodontal disease demonstrated an even stronger association, with 4.67 times higher odds of AMI (95% CI: 2.14-10.19, p < 0.001). Among other variables, hypertension (adjusted OR = 2.31, 95% CI: 1.24-4.31, p = 0.008), diabetes mellitus (adjusted OR = 2.18, 95% CI: 1.12-4.24, p = 0.021) and current smoking (adjusted OR = 2.56, 95% CI: 1.32-4.97, p = 0.005) also remained independently associated with AMI.

## Discussion:

In the current paper, a strong justification is made to believe that there is a strong relationship between the severity of periodontal disease and acute myocardial infarction. The Russell Periodontal Index score of patients with AMI was significantly higher in relation to age and sex matched healthy controls and more than 70 per cent of AMI patients had moderate to severe periodontal disease. Notably, this relationship remained even after the conventional risk factors of cardiovascular diseases had been accounted for, indicating a relationship of independent effect between the periodontal status and acute coronary events. The results are consistent with an increasing literature base on the periodontal-cardiovascular relationship. Epidemiological studies have always depicted high rates of cardiovascular risk among people with periodontal disease [17]. Our results, where the adjusted odds ratio varies between 2.42 and 4.67 with periodontal severity, are not the only instances that have been reported with a 1.5 to 3-fold risk of cardiovascular events to have links with periodontal inflammation [18]. The biological processes of the periodontal- cardiovascular relationship are complex. Chronic periodontal infection is also a continuous source of gram-negative bacteria and their products, such as lipopolysaccharides, that can be transferred into the circulation and provoke inflammatory conditions [19]. It has also been reported that the levels of inflammatory mediators such as C-reactive protein, interleukin-6 and fibrinogen increase in periodontal disease patients and can lead to endothelial dysfunction and rates of atherogenesis [20]. Moreover, periodontal pathogens were directly detected in atherosclerotic plaques, which indicate that oral cavity bacteraemia can be a cause of vascular inflammation and plaque instability [21]. Porphyromonas gingivalis, a periodontal disease keystone pathogen, has been demonstrated to infect endothelial cells, because foam cells and induce platelet aggregation, all of which are essential in atherothrombosis [22].

Periodontal assessment using the Russell Periodontal Index in this study is a standardised procedure that determines the entire range of the severity of the disease. More modern indices, including the Community Periodontal Index and clinical levels of attachment, are more specific, but RPI is still useful in a comparative study because it provides a complete analysis of both inflammatory and destructive periodontal manifestations [23]. The cause-and-effect relationship observed between periodontal severity and the risk of AMI supports the fact that these two factors are connected causally. The participants who had severe periodontal disease showed almost five times the likelihood of AMI relative to the members with healthy or mildly diseased periodontium. This gradient effect agrees with the idea that increased inflammatory load due to increased periodontal devastation has an equal proportionate contribution to cardiovascular risk at the systemic level [24]. There are clinical implications to our findings. Significant periodontal disease is common in AMI patients; therefore, oral health assessment must be included in a general assessment of cardiovascular risks. Moreover, periodontal therapy, which was also indicated to lower systemic inflammatory biomarkers and enhance endothelial functionality, could be a modifiable intervention to reduce cardiovascular risk [25]. Several intervention studies have investigated the impact of periodontal treatment on surrogate markers and cardiovascular outcomes. Systematic reviews have indicated that intensive periodontal therapy results in high levels of C-reactive protein reduction, as well as flow-mediated dilation, which is favourable in terms of vascular effects [26]. Nonetheless, there is limited evidence, based on randomised controlled trials, which indicates a reduction of hard cardiovascular endpoints [27]. The presence of the identified relationship between periodontal disease and AMI could also be a manifestation of similar risk factors and similar pathophysiological mechanisms. The use of smoking, diabetes mellitus and socioeconomic factors are known to be predisposing factors of the two conditions and could be partly descriptive of the observed relationship [28]. Nevertheless, the fact that a strong association remains following statistical control of these confounders is evidence that periodontal disease is an independent risk factor for cardiovascular disease. This study has a cross-sectional nature and therefore, conclusive remarks about causality are not possible. Although there is a strong association between periodontal disease and AMI, it is still possible that there is a shared genetic susceptibility, residual confounding or reverses causation that leads to the observed relationship. This association requires further clarification of its nature in terms of time and causality by conducting prospective cohort studies with recurring periodontal evaluations and subsequent cardiovascular outcomes. The strengths of the study are that periodontal assessment was done by a calibrated examiner, cardiovascular risk factors were fully adjusted and the sample size was large enough with age and sex matched controls. The internal validity of the findings is increased by the fact that there are established diagnostic criteria of AMI and strict exclusion criteria. Methods such as a cross-sectional study design, single-centre location and lack of an inflammatory biomarker will be known as limitations. Also, it is possible that the measurement of periodontal status within the proximity of an AMI is also subject to acute-phase effects, but RPI measures chronic periodontal destruction, which would not be significantly affected by recent events. Long-term follow-up and mechanistic research ought to be incorporated in future research to gain a clearer insight into the periodontal-cardiovascular relationship.

## Conclusion:

We show a significant association between periodontal disease severity and acute myocardial infarction, showing a clear dose-response relationship independent of conventional cardiovascular risk factors. Elevated Russell Periodontal Index scores in AMI patients suggest that periodontal inflammation contributes uniquely to cardiovascular pathology. Thus, we show integrating oral health assessment into cardiovascular risk evaluation and call for longitudinal and interventional studies to confirm causality and preventive potential.
